# INTERNET USE AND LONELINESS: CURE OR CAUSE? LONGITUDINAL ANALYSIS OF OLDER ADULTS’ INTERNET USE

**DOI:** 10.1093/geroni/igz038.051

**Published:** 2019-11-08

**Authors:** Elena Hees, Clemens Tesch-Römer, Oliver Huxhold

**Affiliations:** 1 German Centre of Gerontology, Berlin, Germany; 2 German Centre of Gerontology, Berlin, Berlin, Germany

## Abstract

The internet provides an indispensable platform for social interaction, entertainment and everyday tasks. Especially older adults might benefit from staying engaged online to counteract loneliness. Yet, current research on how internet use effects loneliness still paints a contradictory picture. The current study investigates the longitudinal influence of social internet use forms as opposed to general internet use on loneliness across three years (2014-2017) separately in two age groups (pre-retirement: 40-64 years and post-retirement: 65-85 years), using data from the German Ageing Survey (DEAS). Structural equation modelling shows, that general web use predicts an increase in loneliness in both age-groups. However, contacting friends and family online seems to protect against loneliness over and above the effect of overall internet use, at least for the younger age-group. Therefore, the current study underlines the importance of investigating what exactly people do online instead of seeing the internet as a homogenous tool.

